# Jing Guan Fang, an herbal formula, as an immunomodulator: opposing effects on basal and lipopolysaccharide-induced inflammation of macrophage via JAK/STAT3 and MAPK pathways

**DOI:** 10.3389/fphar.2025.1618488

**Published:** 2025-08-06

**Authors:** Zhi-Hu Lin, Hsin Yeh, Sang-Nguyen-Cao Phan, Li-Lan Liao, Chien-Chang Chen, Wei-Hung Hsu, Chung-Hua Hsu, Tung-Yi Lin

**Affiliations:** ^1^ Institute of Traditional Medicine, National Yang Ming Chiao Tung University, Taipei, Taiwan; ^2^ Department of Ophthalmology, Taipei City Hospital, Taipei, Taiwan; ^3^ Faculty of Traditional Medicine, University of Health Sciences, Vietnam National University, Ho Chi Minh City, Vietnam; ^4^ Department of Chinese Medicine, Taipei City Hospital, Linsen Chinese Medicine and Kunming Branch, Taipei, Taiwan; ^5^ The General Education Center, Ming Chi University of Technology, New Taipei, Taiwan; ^6^ LO-Sheng Hospital Ministry of Health and Welfare, Taipei, Taiwan; ^7^ School of Oral Hygiene, College of Oral Medicine, Taipei Medical University, Taipei, Taiwan; ^8^ School of Chinese Medicine, National Yang Ming Chiao Tung University, Taipei, Taiwan; ^9^ Traditional Chinese Medicine Glycomics Research Center, National Yang Ming Chiao Tung University, Taipei, Taiwan; ^10^ Biomedical Industry Ph.D. Program, National Yang Ming Chiao Tung University, Taipei, Taiwan; ^11^ Research Center for Epidemic Prevention, National Yang Ming Chiao Tung University, Taipei, Taiwan

**Keywords:** Jing Guan Fang, macrophages, immunomodulation, anti-inflammation, lipopolysaccharide

## Abstract

Jing Guan Fang, a formula based on *Forsythia suspensa*, is commonly used for preventing SARS-CoV-2 infection and alleviating cold-like symptoms. However, the precise immunoregulatory mechanisms underlying its effects remain unclear and warrant investigation. This study aims to investigate the immunomodulatory effects of JGF and further elucidate the underlying mechanisms. The results showed that JGF had minimal impact on the cell viability of RAW264.7 and MH-S. In the absence of LPS stimulation, JGF promoted macrophages to produce NO and pro-inflammatory cytokines in a concentration-dependent manner. However, after LPS treatment, the JGF add-on exhibited contrasting effects, with the half-maximal effective concentrations for reducing macrophage-secreted NO and IL-6 being 80 and 180 μg/mL, respectively. Western blot analysis revealed that the JGF supplement marginally induced the production of iNOS and COX-2 without LPS stimulation. However, in LPS-pretreated cells, JGF demonstrated the opposite effect. JGF monotherapy accelerated phosphorylation in the JNK and JAK2 signaling pathways. In contrast, JGF inhibited LPS-stimulated STAT3 phosphorylation by suppressing JNK1/2 activation. Moreover, JGF reduced LPS-induced expression of IL-6 and TNF-α in the lungs and serum of mice. Collectively, the findings suggest that JGF exhibits immunomodulatory activity and suppresses pro-inflammatory cytokine expression caused by LPS.

## Introduction

Traditional herbal medicine, the oldest continual medical practice, has gained worldwide recognition as a complementary therapy due to its favorable outcomes and infrequent side effects (Cyranoski, 2018). Jing Guan Fang (JGF), an herbal formula based on *Forsythia suspense*, has been widely prescribed for illnesses caused by warm pathogens. According to a classical literature Warm Disease Theory of Ye Tian-Shi, warm pathogens indicate exterior heat-related pathogenic factors that enter the superficial portion of the body and later cause a range of hyperthermal diseases, which possibly cover the bacterial infections in the scope of Western medicine. During the COVID-19 outbreak, data disclosed that JGF could alleviate cold-like symptoms and prevent SARS-CoV-2 viral infection (Ping et al., 2022). The underlying mechanisms involve disrupting cell fusion and downregulating angiotensin-converting enzyme-2 (ACE2) and transmembrane serine protease 2 (TMPRSS2). Physicians utilize JGF, known for its anti-inflammatory properties, to restore the health of patients suffering from cold syndrome. Therefore, the immune-related anti-inflammatory efficacy of JGF warrants further validation.

Inflammation is a complex phenomenon that occurs in response to immune-related stimuli, such as injured cells, pathogens, and irritants. Macrophages, the essential innate immune cells, interact with membrane receptors of damaged cells or pathogens, establishing micro-inflammatory sites (Watanabe et al., 2019). LPS-stimulated macrophage models have been increasingly employed to investigate the anti-inflammatory effects of natural products. LPS is widely found in the membranes of most Gram-negative bacteria. These molecules are known to initiate the inflammatory response of macrophages through their interaction with Toll‐like receptors (TLR) (Aderem and Ulevitch, 2000). Evidence shows that macrophage-induced inflammation in COVID-19 supports the release of proinflammatory cytokines, including interferon, TNF-α, IL-1β, IL-6, IL-10, and chemokines (Zheng J. et al., 2021). Remarkably, similar to bacterial infection, SARS-CoV-2 was likely to trigger TLR-mediated inflammation in macrophages (Zheng M. et al., 2021; Zhao et al., 2021). LPS is generally employed to mimic inflammatory responses for identifying potential agents that contribute to suppress symptoms of inflammation.

Many molecules, including nitric oxide (NO), cyclooxygenase-2 (COX-2), and other cytokines, such as interleukin 6 (IL-6), IL-1β, tumor necrosis factor (TNF)-α, are dominant inflammatory markers in immunocyte, especially macrophage (Bogdan et al., 2000). Moreover, mitogen-activated protein kinase (MAPK), Janus kinase/signal transducer and activator of transcription (JAK/STAT), and nuclear factor-kappa B (NF-κB) signaling pathways are critical to inflammatory response in LPS-stimulated RAW264.7 (Ma et al., 2018; Zhou et al., 2017; Lin et al., 2022). Furthermore, STAT3, a crucial downstream signaling moderator, conducts cascades of other proinflammatory mediators (Yu et al., 2009). Another study demonstrated that in SARS-CoV-2 pathophysiology, JNK and JAK/STAT pathways potentially cause inflammation and lung fibrosis after viral infection (Mizutani et al., 2005). In addition, IL-6 is highly related to overt inflammatory reactions in patients with COVID-19 (Mehta et al., 2020; Han et al., 2020). Identifying potent anti-inflammatory agents may improve inflammation-related discomfort in patients with COVID-19 or cold.

Sore throat is a common manifestation among patients with COVID-19 or cold-like syndromes. Prior studies showed that JGF could ameliorate sore discomfort (Ping et al., 2022). Nevertheless, the anti-inflammatory mode of action of JGF remains unknown. In this study, NO and IL-6 were utilized to evaluate the anti-inflammatory effects of JGF in LPS-stimulated macrophages. The potential mechanisms involved in the anti-inflammatory process of JGF were further investigated. The current findings suggest that JGF could barely activate macrophages under normal condition, however, it could effectively suppress LPS-induced NO and IL-6 secretion by inhibiting the JNK1 and STAT3 pathway.

## Materials and methods

### JGF preparation

**SCHEME 1:**
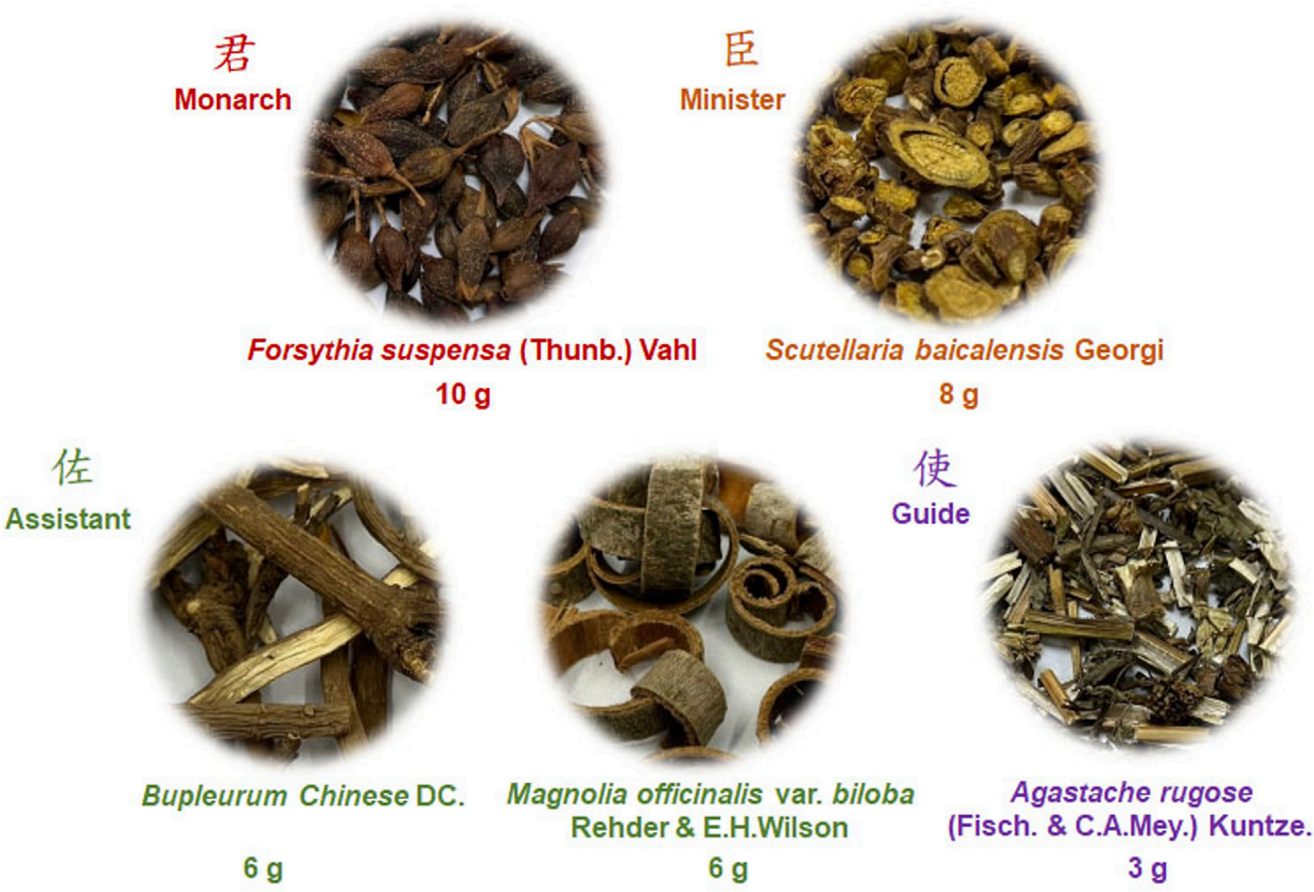
Compositions of JGF.

JGF composition was manufactured by the Branch of Linsen Chinese and Kunming, Taipei City Hospital (Taipei, Taiwan). JGF consists of five medicinal herbs (Scheme 1): 10 g of *Forsythia suspensa* (Thunb.) Vahl (Monarch; 君; cat no.: 057A-0), 8 g of *Scutellaria baicalensis* Georgi (Minister; 臣; cat no.: 051-1), 6 g of *Bupleurum Chinese* DC. (Assistant; 佐; cat no.: 009-1), 6 g of *Magnolia officinalis* var. *biloba* Rehder & E.H. Wilson (Assistant; 佐; cat no.: 085K-1) and 3 g of *Agastache rugose* (Fisch. & C.A. Mey.) Kuntze (Guide; 使; cat no.: 089-1). All medicinal herbs were purchased from the GMP-certified pharmaceutical company (Sun Ten Pharmaceutical Co., Ltd. Taiwan). The preparation and quality control of JGF followed those in a previous study (Ping et al., 2022). Briefly, after decoction, the water-extract was filtered through filter paper to remove insoluble residues, then pre-frozen at −80°C overnight, followed by vacuum freeze-drying. The HPLC fingerprint profile of JGF from Sun Ten Pharmaceutical Co., Ltd. was established using reverse phase-HPLC with a diode-array detector (Supplementary Figure S1).

### Cell lines

Murine macrophage-like RAW264.7 and alveolar macrophage MH-S cells were purchased from the Bioresource Collection and Research Center (BCRC, Hsinchu, Taiwan). RAW264.7 and MH-S cells were respectively cultured in Dulbecco’s modified Eagle’s medium (DMEM, GIBCO-Life Technologies) and Roswell Park Memorial Institute (RPMI) 1640 medium (GIBCO-Life Technologies) at 37°C under a mixture of 95% air and 5% CO_2_. The medium was supplemented with 5% heat-inactivated fetal bovine serum (FBS, HyClone, Marlborough, MA), 3.7 g/L NaHCO_3_, 100 units/mL penicillin and 100 units/mL streptomycin (Biological Industries, Cromwell, CT). Attached RAW264.7 and MH-S cells were detached using Trypsin-EDTA (Invitrogen, Co., Carlsbad, CA).

### Cell viability assay

The viability of RAW264.7 and MH-S cells was measured using the crystal violet assay as in previous studies (Ping et al., 2022; Lin et al., 2023). Initially, cells were seeded in 12-well plates at a density of 5 × 10^4^ cells per well and incubated overnight. Then, cells were randomly inoculated with or without LPS (1 μg/mL). Later, various concentrations of JGF (0–600 μg/mL) were added to the cell cultures for 24 h. After treatment, suspended cells were washed with phosphate-buffered saline (PBS). Then, the attached (live) cells were stained with 1% crystal violet (dissolved in 30% ethanol) for 1 h and removed. Next, cells were washed with deionized water, dried and dissolved with 30% acetic acid to release the crystal violet for detection. The ELISA reader was used to measrure the O.D (optical density) at the absorbance wavelength of 570 nm.

### Determination of NO production

RAW264.7 and MH-S cells were cultured in 96-well plates at a density of 1 × 10^5^ cells/well and stimulated with LPS (100 ng/mL; *E. coli* O55:B5; Sigma Chemical L 2630, St. Louis, MO, USA) in the presence or absence of JGF at different concentrations (0–600 μg/mL) for 24 h. NO production was determined using the Griess assay as described previously (Lin et al., 2023). Griess reagent was added to the culture medium, followed by a 10-min incubation. A standard curve was generated using NaNO_2_. Finally, absorbance was measured at 540 nm using a microplate reader. In each experiment, the amount of NO produced by LPS stimulation was defined as 100%.

### Measurement of IL-6 and TNF-α

RAW264.7 and MH-S cells were seeded in 96-well plates at a density of 2 × 10^4^ cells/well for 24 h. The cells were treated with various concentrations of JGF (0–600 μg/mL) and/or LPS (100 ng/mL) for another 24 h. The supernatants were collected for measuring IL-6 and TNF-α concentrations using an enzyme-linked immunosorbent assay (ELISA) kit (BioLegend, San Diego, CA, USA) as described previously (Lin et al., 2023). The optical densities, indicative of IL-6 and TNF-α concentrations, were measured by TECAN Sunrise TMELISA Reader (Tecan Group Ltd., Männedorf, Switzerland) at wavelengths of 450 nm and 550 nm (reference absorbance). The levels of IL-6 and TNF-α after treatment with LPS alone were designated to be 100%.

### Cytokine array

MH-S cells were seeded in 6-well plates at a density of 5 × 10^5^ cells/well for 24 h, and then incubated in the fresh medium containing only JGF (600 μg/mL) or LPS (100 ng/mL), or JGF + LPS for another 24 h. After stimulation, the supernatants were collected and centrifuged at 1,000 × g for 5 min at 4°C, followed by cytokine array (C3 and C4) analysis (RayBiotech Life, Inc. GA 30092, USA).

### Western blot assay

Cells were cultured in 6-cm dishes (Corning, USA) at a density of 5 × 10^5^ cells/well and divided into groups of cells treated with JGF (200 μg/mL) alone, and LPS (100 ng/mL) plus JGF (200 μg/mL). After treatment, cells were rinsed with cold PBS and lysed in a specific lysis buffer (Yeh et al., 2022). Then, whole cell lysates were centrifuged (13,000 × g, 10 min, 4°C) and the supernatants were collected for Western blot analysis. The supernatants were quantified using Bradford assay (Bio-Rad, Hercules, CA). The cell extracts (21 μg) were then separated by 10% sodium dodecyl sulfate–polyacrylamide gel electrophoresis (SDS-PAGE) and transferred to PVDF (0.22 μm) membranes. The PVDF membranes were blocked with 10% bovine serum albumin (60 min, room temperature). After blocking, samples were incubated at 4°C overnight with the primary antibodies mentioned below. The indicated molecules were then detected using secondary antibodies. Antibodies against iNOS, p-JAK2 (Tyr1007/1008), p-STAT3 (Tyr705), and β-actin antibodies were purchased from GeneTex, Inc. (Hsinchu, Taiwan). Anti-p-ERK1/2, p-P38 and p-JNK1/2 (Thr183/Tyr185) antibodies were purchased from Cell Signaling Technology, Inc. (MA, USA). Anti-COX-2 antibody was purchased from Santa Cruz Biotechnology, Inc. (TX, USA). In this experiment, blots were visualized using GE Amersham Imager 600. β-actin and α-tubulin expressions act as internal controls.

### LPS-induced acute inflammatory mouse model

Male C57BL/6 mice (8–10 weeks old) were purchased from the National Laboratory Animal Center (Taipei, Taiwan) and nurtured at the animal facility of the National Yang Ming Chiao Tung University. The LPS-induced inflammatory animal model mouse model was modified as described previously (Ou et al., 2023; Nguyen et al., 2021). As recommended by TM experts, the dose of JGF for humans was 30 mg/kg orally, and the calculated oral dose for mice was approximately 370 mg/kg. JGF (400 mg/kg) was administered orally (p.o.) 1 h before as well as 1 and 6 h after intraperitoneal injection (i.p.) of LPS (20 mg/kg). JGF was prepared in deionized water (ddH_2_O), and LPS was prepared in normal saline and filtered through a 0.22 μm membrane. Mice were allocated (n = 5 per group) to groups of CTL (ddH_2_O + saline), LPS (ddH_2_O+ LPS 20 mg/kg), JGF (JGF 400 mg/kg + saline), and JGF + LPS (JGF 400 mg/kg + LPS 20 mg/kg). Blood collection was at 1 h post-LPS injection for IL-6 and TNF-α measurement. At 12 h post-LPS injection, the mice were weighed and then sacrificed using carbon dioxide. Serum, bronchoalveolar lavage fluid (BALF) and abdominal fluid were gathered for the IL-6 TNF-α measurement.

### Statistical analysis

The statistical differences between the mean values of each group were analyzed by one-way ANOVA with post-hoc Tukey tests and t-test using *GraphPad Prism 8.4*. P values less than 0.05 were regarded as statistically significant. Each experiment was repeated three times or as indicated. All data are expressed as mean ± standard deviation (SD).

## Results

### Effects of JGF on cell viability of macrophages, RAW264.7, and MH-S cells

**FIGURE 1:**
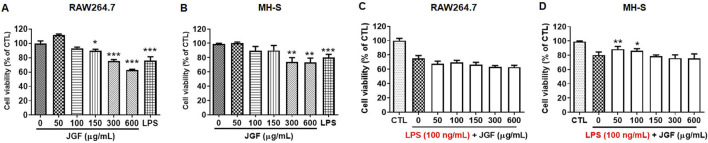
JGF slightly inhibits cell viability of macrophages. **(A,B)** RAW264.7 **(A)** and MH-S **(B)** cells were treated with JGF at various concentrations (50–600 μg/mL) for 24 h. **(C,D)** RAW264.7 **(C)** and MH-S **(D)** cells were stimulated with LPS (100 ng/mL) in the presence or absence of JGF (0–600 μg/mL) for 24 h simultaneously. The viability of macrophage was determined using crystal violet assay. Cell viability of each JGF-LPS treatment group was normalized against an untreated control (CTL). Significant differences are shown (^***^P < 0.001, compared with the untreated group).

To examine the immunomodulatory effect, RAW264.7 and MH-S were treated with various concentrations of JGF (0–600 μg/mL) for 24 h. As shown in Figures 1A,B, JGF showed insignificant cytotoxicity to either cell line except at a high concentration (600 μg/mL), at which JGF eradicated 35% and 20% of RAW264.7 and MH-S cells, respectively. The half-maximal inhibitory concentration (IC_50_) of JGF in those cells exceeded 600 μg/mL. Subsequently, the anti-inflammatory activity of JGF on LPS-stimulated macrophages was evaluated. These cell cultures were co-treated with various concentrations of JGF (0–600 μg/mL) and LPS (100 ng/mL) for 24 h. JGF could not sustain the survival rate under LPS stimulation, while low concentrations of JGF (50 and 100 μg/mL) rescued slightly MH-S cell viability (Figures 1C,D).

### Effects of JGF on immunomodulatory molecules profiles of macrophages

**FIGURE 2:**
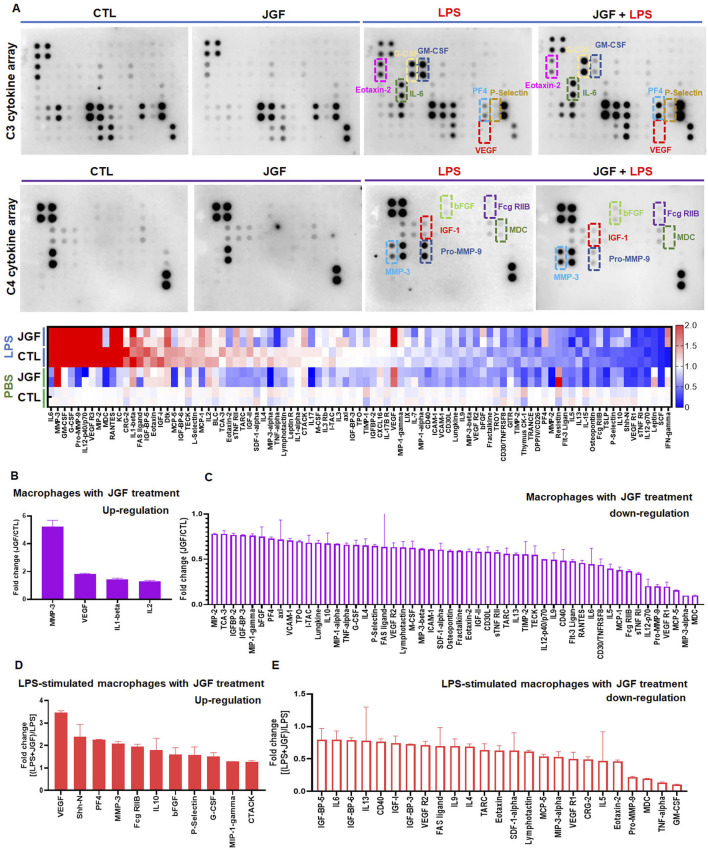
JGF induces changes in cytokine profiles of MH-S cells. Macrophages (MH-S) were treated with JGF (600 μg/mL), LPS (100 ng/mL), or a combination of LPS and JGF and cultured for 24 h. The culture supernatants were then collected and analyzed directly using a cytokine array. Quantitative analysis was performed using ImageQuant™ TL analysis software. **(A)** Up panel: chemiluminescent images of cytokine arrays produced by MH-S macrophages treated with CTL, JGF, LPS, or LPS + JGF. Down panel: The heatmap of JGF-mediated cytokine profiles on MH-S cells with and without LPS treatment. **(B,C)** Quantitative graph shows upregulation **(B)** or downregulation **(C)** of cytokine secretion by MH-S macrophages in response to JGF treatment. **(D,E)** Quantitative graph illustrates LPS-induced inflammatory cytokine secretion by JGF in MH-S macrophages.

To evaluate the immune response of macrophages to JGF, we performed an array to determine the cytokine profiles in macrophages with or without LPS stimulation. The signal intensities were extracted and normalized (Supplementary Tables S1, S2). The indicated cytokine expressions varied among groups. More than 50 molecules exhibited altered expression patterns in JGF-treated MH-S macrophages (Figures 2A,C). Notably, in the JGF (600 μg/mL) treatment group, cytokines, including MMP-3, VEGF, IL-1β, and IL-2, were visibly elevated compared with the control (CTL) group (Figures 2A,B). However, macrophage-derived chemokine (MDC), macrophage inflammatory protein-3 Alpha (MIP-3α), and monocyte chemotactic protein-5 (MCP-5), which amplify the host response to inflammation (Katou et al., 2001; Dieu-Nosjean et al., 2000; Sarafi et al., 1997), were dramatically reduced following JGF treatment (Figure 2C). Additionally, pro-inflammatory factors such as TNF-α, IL-6, IL-12, MIP-1α were downregulated in MH-S cells after JGF treatment, suggesting that JGF may exhibit immunomodulatory properties in resting macrophages.

Contrary to the CTL group, pro-inflammatory cytokines such as IL-6, GM-CSF, G-CSF, Pro-MMP-9, IL-12, VEGFR3, MIP-2, MDC, RANTES, KC, CRG-2, and IL-1β were significantly expressed after LPS (100 ng/mL) treatment (Figure 2A), confirming LPS as a potent inflammatory stimulus. Interestingly, VEGF levels were further elevated in LPS-stimulated macrophages upon JGF treatment (Figure 2D), suggesting that JGF may influence LPS-induced macrophage polarization (Lai et al., 2019; Wheeler et al., 2018). We further the effects of JGF on LPS-induced pro-inflammatory molecules. As shown in Figure 2E, JGF countered the LPS detrimental effect by decreasing GM-CSF, TNF-α, MDC, and Pro-MMP-9 (Katou et al., 2001; Parajuli et al., 2012; Wang et al., 2009). In short, these findings demonstrated that JGF did modulate the expression of cytokines in macrophages and may function as a potent immunomodulatory agent.

### Effects of JGF on modulation of immune-related signaling pathways

**FIGURE 3:**
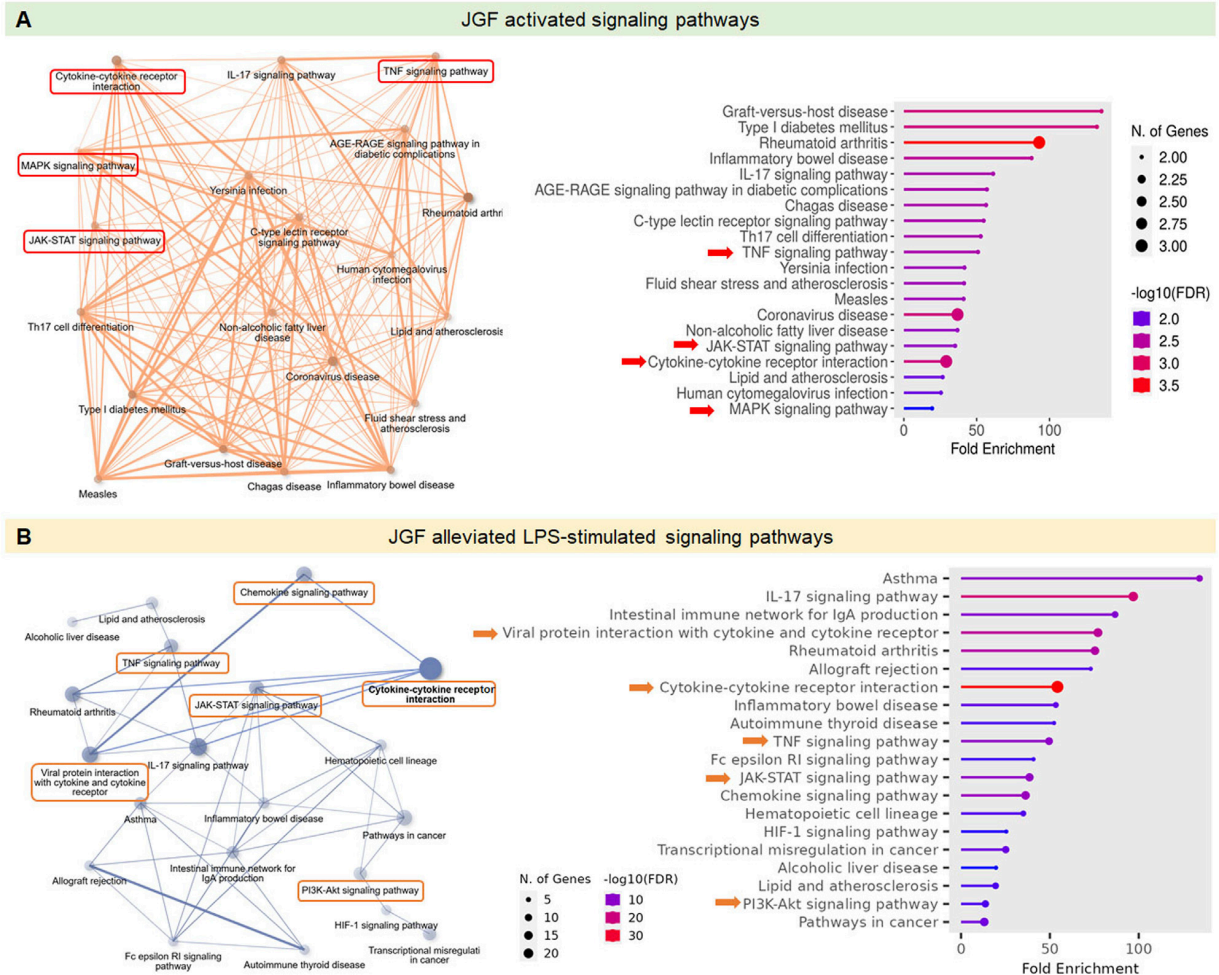
Effects of JGF on modulation of immune-related signaling pathways. The GO and KEGG pathway analysis was employed to explore the immunoregulatory profiles of JGF under both basal and LPS-induced inflammatory conditions. **(A)** Signaling pathways activated by JGF under basal conditions. **(B)** Signaling pathways suppressed by JGF in response to LPS stimulation. ShinyGO 0.82 was used to plot the top 20 KEGG analysis results and displayed as an enriched dot bubble and bar plot.

We further used KEGG (Kyoto Encyclopedia of Genes and Genomes) and GO (Gene Ontology) platforms to investigate the immunoregulatory profiles of JGF under basal and inflammatory conditions. As shown in Figure 3A, the enrichment network and dot plot showed that JGF activated a wide range of immune and inflammatory signaling pathways. Notably, the cytokine–cytokine receptor interaction, IL-17 signaling, and TNF signaling pathways were significantly enriched. Additional intracellular pathways such as the MAPK and JAK-STAT signaling pathways were upregulated, suggesting that JGF may broadly enhance innate immune signaling. In contrast, the key pro-inflammatory pathways, including cytokine–cytokine receptor interaction, TNF, IL-17, JAK-STAT, and PI3K-AKT pathways, were downregulated in the immunosuppressive role of JGF under LPS-induced inflammatory stress (Figure 3B). Interestingly, viral protein interaction with cytokine and cytokine receptor was shown in JGF alleviated LPS-mediated signaling, which may be correlated to JGF as an agent for COVID-19 (Ping et al., 2022). Together, JGF may function as a bidirectional immune modulator.

### Effects of JGF on LPS-stimulated inflammatory molecules of macrophages

**FIGURE 4:**
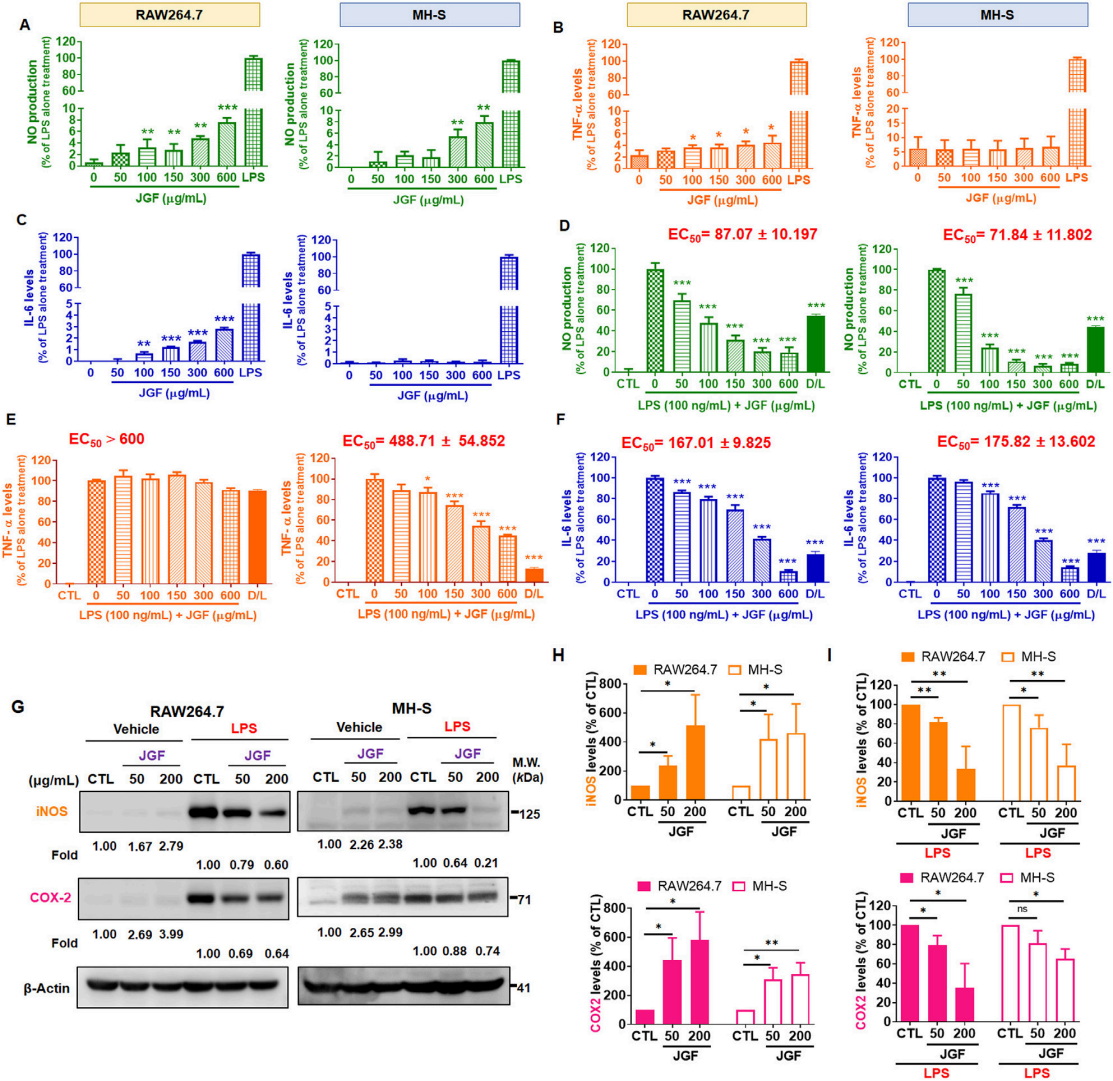
JGF reduces LPS-induced pro-inflammatory molecules of macrophages. **(A–C)** RAW264.7 and MH-S cells were treated with various concentrations of JGF (50–600 μg/mL) and LPS (100 ng/mL; as positive control) for 24 h. **(D–F)** RAW264.7 and MH-S cells were stimulated with LPS (100 ng/mL) in the presence or absence of JGF (0–600 μg/mL) for 24 h simultaneously. **(A,D)** NO levels. **(B,E)** TNF-α levels. **(C,F)** IL-6 levels. The D/L group (20 µM dexamethasone + 100 ng/mL LPS) as a positive control for comparison. Each JGF-treated group was normalized against the control (LPS-alone treatment) group. EC_50_ of JGF was calculated using *GraphPad Prism 8.4*. The data were representative of three separate experiments and were presented as the mean ± SDs; the error bars indicated SD. Significant differences are shown (^***^P < 0.001, compared with the untreated or LPS-alone treatment group). **(G–I)** RAW264.7 and MH-S cells were stimulated with or without LPS (100 ng/mL) in the presence or absence of JGF (50 and 200 μg/mL) for 24 h simultaneously. **(G)** The expressions of iNOS and COX-2 were determined using Western blot. β-actin was used as the internal control. The densitometry of indicated proteins was quantified using the ImageJ software. Each JGF-treated group was normalized against the control (CTL) group. Moreover, each JGF-LPS co-treatment group was normalized against the LPS-alone treatment group. **(H,I)** The results from Figure 4G are presented as means ± SDs (n = 3), and the figure shows significant differences (*P < 0.05, **P < 0.01) compared with the control group.

JGF exhibited different macrophagic responses. Induction of NO, TNF-α, and IL-6 by LPS served as markers of inflammatory activation in these cells (Guha and Mackman, 2001; Greenhill et al., 2011). To assess whether JGF itself triggers an inflammatory phenotype, macrophages were treated with JGF alone. As shown in Figure 4A, both Griess and ELISA assays revealed that JGF upregulated NO levels in RAW264.7 and MH-S cells concentration-dependently. However, even at the highest concentration tested (600 μg/mL), JGF-induced NO levels remained approximately ten-fold lower than those elicited by LPS (100 ng/mL). IL-6 and TNF-α expressions in RAW264.7 were slightly elevated by JGF administration, whereas MH-S cells showed trivial change (Figures 4B,C). To investigate the potential involvement of MAPK signaling pathways in JGF and LPS-induced NO production, three specific MAPK inhibitors were used: PD98059 (ERK inhibitor), SP600125 (JNK inhibitor), and SB203580 (p38 inhibitor). As shown in Supplementary Figure S2, although JGF-induced NO production was relatively low (approximately 10% of LPS-induced levels), individual MAPK inhibitors showed slight suppressive effects. Notably, the combination of all three inhibitors resulted in a more pronounced inhibition. These results suggested that JGF may provoke innate immune adaptation (Figure 3A).

In addition, JGF had a broad inhibitory effect on LPS-induced inflammatory factors (Figure 2). Therefore, we deciphered whether JGF could reverse NO, TNF-α, and IL-6 changes in macrophages pre-treated with LPS. As shown in Figure 4D, JGF significantly decreased NO emission from LPS-stimulated macrophages in a concentration-dependent manner. The half-maximal effective concentration (EC_50_) of JGF against LPS-induced NO production on RAW264.7 and MH-S cells were 87.07 and 71.84 μg/mL, respectively. JGF also inhibited TNF-α secretion on LPS-stimulated MH-S cells but scarcely impacted RAW264.7 (Figure 4E). The EC_50_ to TNF-α on MH-S cells was 488.71 μg/mL. Correspondingly, JGF reversed the effect of LPS on the IL-6 level (Figure 4F), in which the EC_50_ for RAW264.7 and MH-S cells were 167.01 and 175.82 μg/mL, respectively. Thus, despite slightly activating inflammatory markers when solely administered, JGF could adequately inhibit the acute LPS-mediated reactions in macrophages.

### Effects of JGF on LPS-induced iNOS and COX-2 of macrophages

As mentioned, JGF inhibited NO production in LPS-stimulated RAW264.7 and MH-S cells. The biological effects of JGF on pro-inflammatory mediators were examined using Western blot assay. iNOS and COX-2 are two critical molecules involved in the pathophysiology of inflammation (Surh et al., 2001). As shown in Figure 4G, JGF increased slightly the quantity of iNOS and COX-2 compared with that of LPS. Specifically, JGF-induced COX-2 expression more significantly in RAW264.7 cells than in MH-S cells, which may indicate why JGF induced less inflammatory responses in MH-S cells as shown in Figure 4H. On top of that, JGF repressed the intense expression of iNOS and COX-2 in LPS-stimulated RAW264.7 and MH-S cells (Figure 4I). Together, besides mildly enhancing immune ability, JGF significantly reduced LPS-driven overt inflammation.

### Effects of JGF on LPS-induced intracellular molecules of macrophages

**FIGURE 5:**
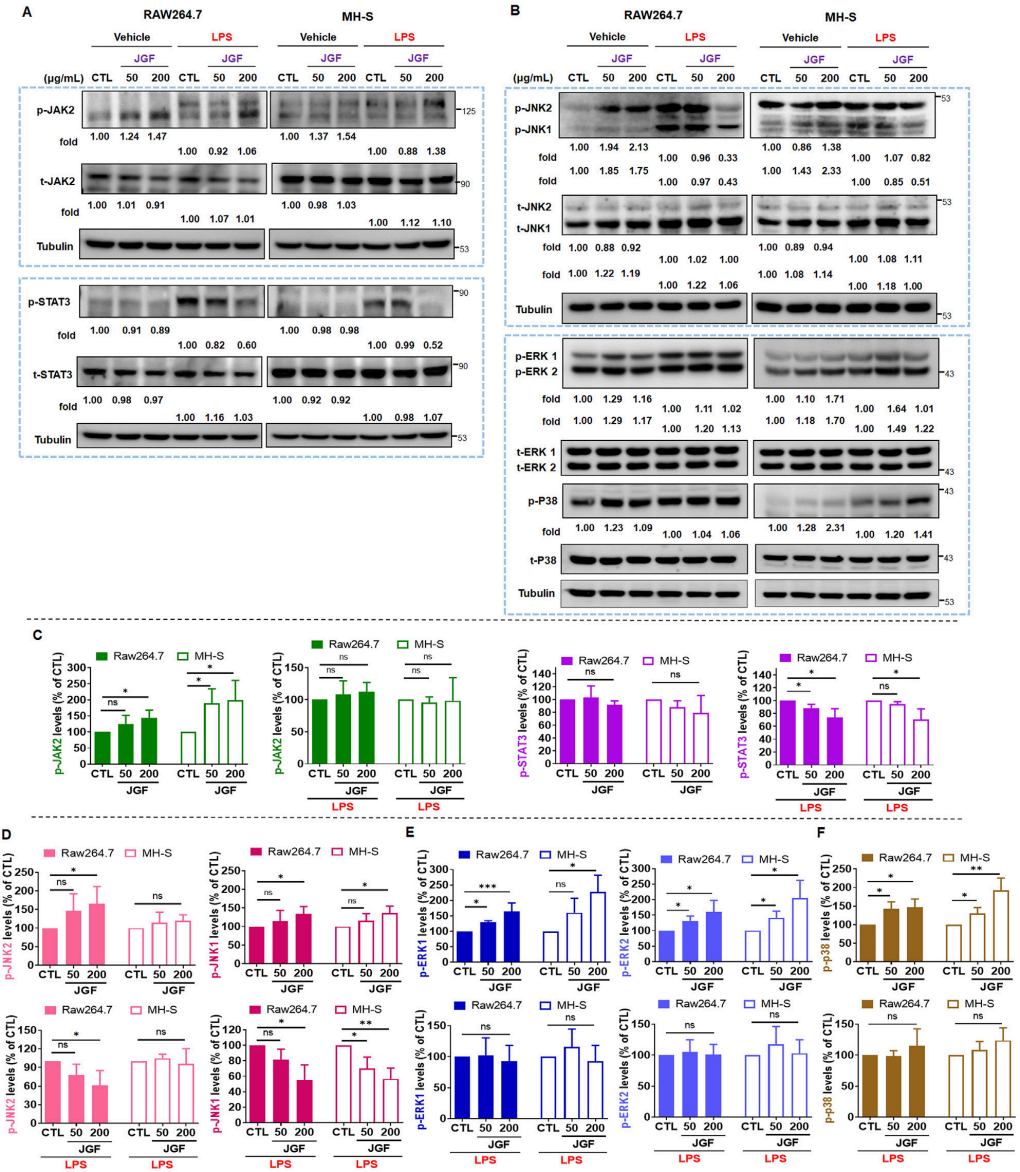
JGF inhibits phosphorylation of inflammatory molecules on macrophages. **(A,B)** RAW264.7 and MH-S cells were stimulated with or without LPS (100 ng/mL) in the presence or absence of JGF (50 and 200 μg/mL) for 24 h simultaneously. The phosphorylated levels of JAK2, STAT3, JNK1/2, ERK1/2, P38, and total levels of JAK2, STAT3, JNK1/2, ERK1/2 and P38 were determined using Western blot. Tubulin were used as internal controls. The densitometry of indicated proteins was quantified using the ImageJ software. Each JGF-treated group was normalized against the control (CTL) group. Moreover, each JGF-LPS co-treatment group was normalized against the LPS-alone treatment group. **(C–F)** The results from Figures 6A,B are presented as means ± SDs (n = 3), and the figure shows significant differences (*P < 0.05, **P < 0.01) compared with the control group.

The JAK/STAT pathway plays a significant role in cellular response to LPS toxification (Lin et al., 2022). To explore the potential intracellular effects of JGF on RAW264.7 and MH-S cells, both in the presence and absence of LPS stimulation, the activation status of JAK/STAT signaling was examined. As illustrated in Figure 5A, without LPS stimulation, JGF upregulated the JAK2 phosphorylation (Figure 5C) but has no significant effect on the activation of STAT3 (Figure 5D). Interestingly, in LPS-stimulated cells, co-treatment with JGF resulted in a marked reduction in STAT3 phosphorylation. The MAPK pathway, comprising JNK1/2, ERK1/2, and p38 cascades, is known to play a critical role in LPS-induced inflammatory responses (Kyriakis and Avruch, 2012). We first examined the phosphorylation of JNK1/2. As shown in Figures 5B,D, JGF induced phosphorylation of JNK1/2—particularly JNK1—in the absence of LPS. However, in the presence of LPS, JGF significantly suppressed JNK1 activation. In contrast, JGF did not affect JNK2 phosphorylation in MH-S cells. We further showed that inhibition of JNK effectively inhibited the phosphorylation of STAT3 induced by LPS stimulation in macrophages (Supplementary Figure S3). In addition, while JGF enhanced ERK1/2 and p38 phosphorylation under basal conditions, it failed to suppress their LPS-induced activation (Figures 5E,F). These findings suggest that JGF may activate macrophages under non-inflammatory conditions, yet mitigate excessive inflammatory responses triggered by LPS.

### Effects of JGF on LPS-induced acute inflammation in mice

**FIGURE 6:**
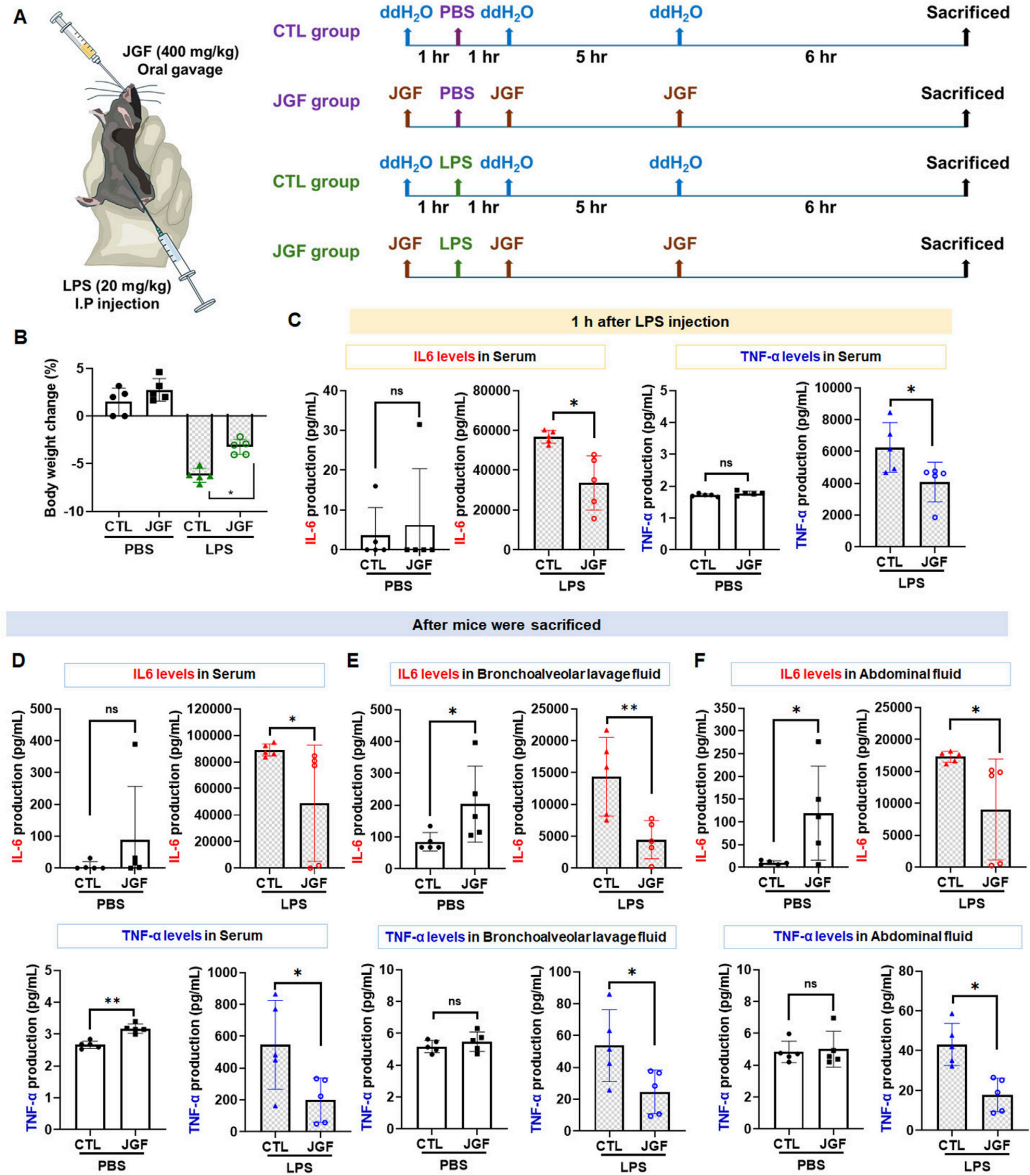
**(A)** Experimental flow chart. JGF (400 mg/kg) was administered orally (p.o.) 1 h before intraperitoneal injection (i.p.) of LPS (20 mg/kg), as well as 1 h and 6 h after injection to mice (n = 5). At 12 h of post-LPS injection, the mice were weighed. After the sacrifice of mice by CO2, bronchoalveolar lavage fluid (BALF) and abdominal fluid were collected for IL-6 and TNF-α measurement. **(B)** Body weight changes (%) in the CTL mice and LPS-treated mice without or with the treatment of JGF were recorded at sacrifice. **(D-F)** The protein levels of IL-6, 1 hour after LPS injection in the serum **(C)**, after mice sacrifice in serum **(D)**, in BALF (E), and abdominal fluid **(F)** of the CTL mice and LPS-treated mice without or with the treatment of JGF were analyzed using ELISA. Each experimental group consisted of five animals (n = 5). The LPS dosage was 20 mg/kg (i.p.) and the JGF dosage was 400 mg/kg (p.o.). The asterisk indicates significant differences from the LPS-treated (*P < 0.05).

LPS-induced endotoxemia is a well-accepted *in vivo* model for conducting systemic inflammatory response syndrome (SIRS), leading to numerous lethal consequences in animals and increases in pro-inflammatory cytokines (Seemann et al., 2017). Accordingly, we established an LPS-challenged mouse model to assess the anti-inflammatory potential of JGF (Figure 6A). JGF treatment markedly mitigated body weight loss in LPS-exposed mice, with a relative improvement of approximately 3% (Figure 6B). In general, IL-6 and TNF-α are crucial mediators for regulating local and systemic acute inflammation (Nandi et al., 2010). To evaluate the effect of JGF on these cytokines, serum levels of IL-6 and TNF-α were initially measured. As shown in Figure 6C, LPS administration significantly increased circulating levels of both cytokines, whereas JGF treatment led to reductions of 41% and 35% in IL-6 and TNF-α, respectively. Notably, JGF alone did not elicit significant cytokine induction in serum after 1 hour of administration (Figure 6C). Interestingly, even after three administrations of JGF, serum IL-6 levels in mice remained largely unchanged, while TNF-α levels showed a slight increase (Figure 6D). Nevertheless, the overall expression of both cytokines remained low. Given that acute inflammation can progress to multi-organ dysfunction, particularly affecting the lungs and peritoneal cavity, IL-6 and TNF-α levels in bronchoalveolar and abdominal fluid samples were further measured (Chen et al., 2018). We found that JGF alone treatment could induce IL-6 levels but not TNF-α in both compartments (Figures 6E,F), which was consistent with our *in vitro* findings. Intriguingly, in mice challenged with LPS, subsequent treatment with JGF resulted in substantial decreases in IL-6 and TNF-α levels in lung and peritoneal fluids at 12 h, with reductions of 68% and 54% for IL-6, and 48% and 59% for TNF-α, respectively (Figures 6E,F). Together, these results indicated that JGF functions as an immunomodulatory agent predominantly by modulating macrophage activity in mice.

## Discussion

JGF, an herbal decoction based on *F. suspensa*, has showed potential therapeutic benefits against COVID-19. During the pandemic, it was reported to help prevent SARS-CoV-2 infection and alleviate cold-related symptoms, such as sore throat (Ping et al., 2022). Pain sensation could be ameliorated by lessening the degree of inflammation (Eccles, 2023). Even with the aforementioned efficacy, the underlying mechanism of JGF on excessive inflammation has yet to be determined. This study manifested that upon LPS-stimulated macrophages, JGF significantly diminished the production of NO, TNF-α, and IL-6. In particular, JGF reduced LPS-induced NO and IL-6 production by more than 50%. Compared to dexamethasone (a clinical drug), high concentration (600 μg/mL) of JGF exhibits higher ant-inflammatory activity. Further profiling of cytokine expression revealed distinct changes in macrophage response upon treatment with JGF alone or in combination with LPS (Figures 2, 3). Mechanistically, JGF activated the JAK2 and MAPK signaling pathways, contributing to macrophage activation. Notably, JGF inhibited LPS-activated STAT3 and JNK1 phosphorylation, leading to a significant downregulation of iNOS and COX-2 levels. In brief, JGF exerts anti-inflammatory effects by modulating key signaling pathways, which may underlie its clinical benefits. Nonetheless, large-scale randomized clinical trials are warranted to further validate the efficacy and therapeutic value of JGF.

Previous studies have established the anti-inflammatory properties of the components in JGF (Supplementary Table S3) (Gong et al., 2021). For example, forsythin from *F. suspensa (Thunb.) Vahl,* or Lianqiao, the chief herb, could suppress LPS-induced inflammation by subduing the signals of JAK-STAT and p38/MAPK in macrophage cytoplasm (Pan et al., 2014). Another primary herb, Huangqin (*S. baicalensis* Georgi), potentially degrades the protein expressions of iNOS and COX-2 in LPS-stimulated RAW264.7 cells (Chen et al., 2017). Furthermore, evidence showed that two major compounds (baicalin and wogonin-7-O-glucuronide) from Huangqin not only are non-toxic to macrophages but also reduce the production of LPS-induced pro-inflammatory factors via inhibiting the STAT3 pathway (Supplementary Figure S4), reflecting the biological activity of JGF. In addition, some studies revealed that both *B. Chinese* DC. and *M. officinalis var. biloba* Rehder & E. H. Wilson possess an inhibitory effect on the NF-κB signaling pathway (Song et al., 2017; Chunlian et al., 2014). As an assistant ingredient, *A. rugosa* (Fisch & C. A. Mey) shows exquisite anti-inflammatory activity and is considered a natural source for inflammatory diseases (Nan et al., 2022). Albeit with the aptitude for relieving inflammation of each herb, researchers are still looking for how the herbal consolidation manipulates the macrophage-regulated inflammation. The herb-herb interaction might boost the synergistic effect and attenuate toxicity, which makes the polyherbal formula more beneficial (Bu et al., 2020; Liao et al., 2021; Bui et al., 2019). This study is the first to verify that JGF could alleviate the inflammation induced by LPS in macrophages.

**FIGURE 7:**
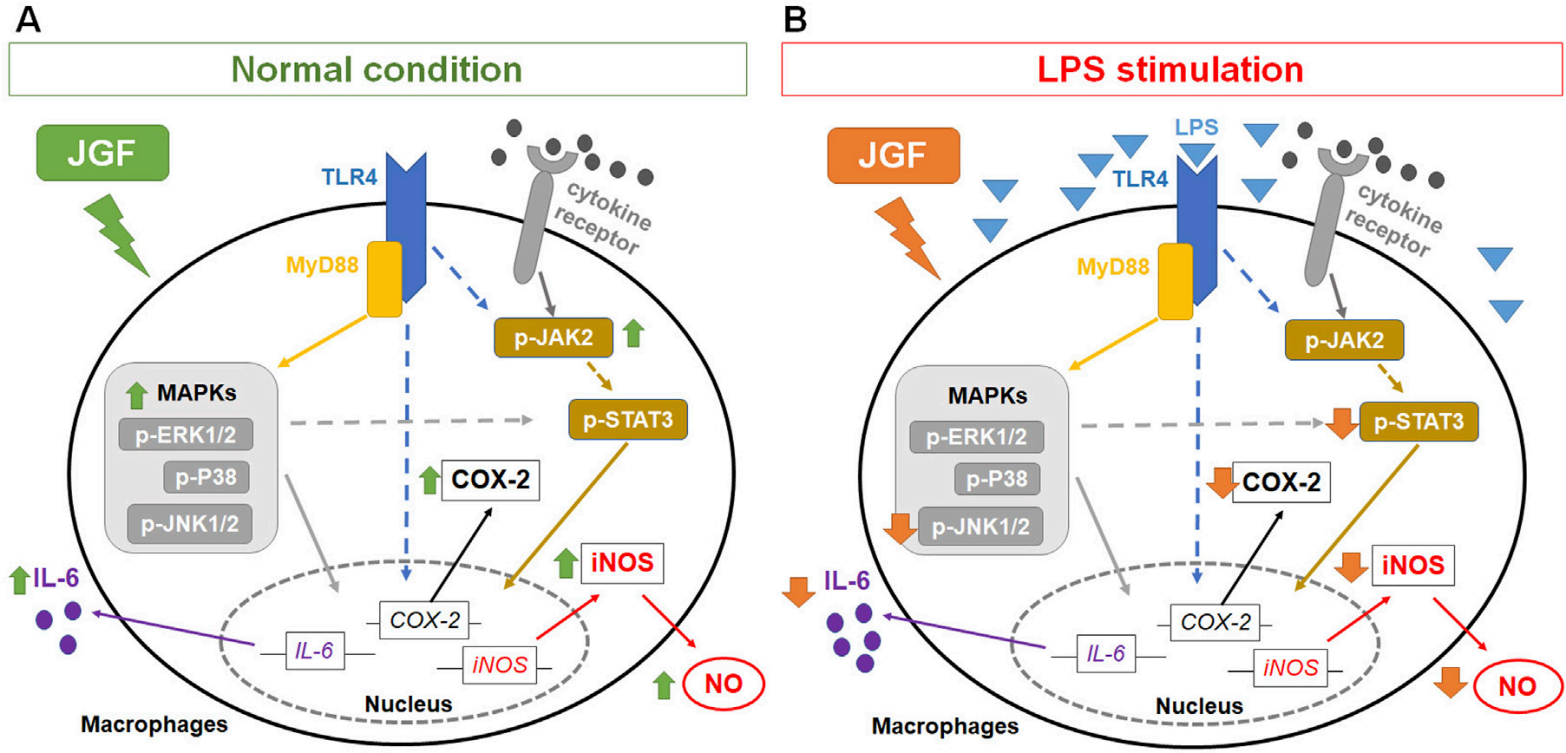
Potential mechanisms of JGF on macrophage-based immunomodulation. The potential mechanism of JGF on activation of macrophages **(A)** and inhibition of LPS-induced inflammatory responses **(B)**.

Immunomodulation is one of the intrinsic properties that herbal formulas possess for health improvement. Acknowledging the inflammatory scheme in macrophages would reflect how medicinal herbs could adjust the immune system. The present experiment showed that JGF induced the production of proinflammatory molecules in macrophages without LPS stimulation (Figures 2, 3). Moreover, we found that JGF induced a slight increase in phagocytic activity at higher concentrations (Supplementary Figure S5). The probable mechanism might be via upregulation of JAK2 and MAPK-mediated iNOS/COX-2 pathway (Figures 5–7), suggesting that JGF may exhibit immune activation under normal conditions. Moreover, in the NF-κB-related pathway, JGF did not affect phosphorylation of the P65 protein in macrophages with and without LPS stimulation (data not shown). Interestingly, we found that JGF downregulated expressions of Toll-like receptors, especially under LPS-induced conditions (data not shown). In the future, the mechanism of JGF and its components-triggered these cellular responses should be further examined. On the other hand, the cytokine array showed that JGF alleviated LPS-induced acute inflammatory molecules such as GM-CSF, IL-6, and pro-MMP-9 (Figure 2). Specifically, GM-CSF could attract macrophages to migrate to the inflammation site and cause a series of chain reactions, including increasing IL-6, TNF-α and MMP9, to aggravate inflammation (Li et al., 2014). Of note is that JGF could significantly reduce LPS-induced GM-CSF production, meaning that JGF may interrupt the infinite loop of inflammation amplification, in which macrophage plays a central role. The ability of JGF to simultaneously dampen these key inflammatory axes suggests a strong context-dependent immunomodulatory mechanism. Given the solid anti-inflammatory activity observed in LPS-stimulated alveolar macrophage cells, JGF could mitigate macrophage-mediated inflammation in lung tissue.

GM-CSF boosts the LPS-induced expression of pro-inflammatory mediators through the NF-κB, MAPK and STAT3 pathways (Bozinovski et al., 2002; Liu et al., 2018; Beurel and Jope, 2009). The cytokines also possess positive feedback in restimulating GM-CSF, which assembles an infinite loop (Shi et al., 2006). Moreover, STAT3 is also a crucial downstream signal mediator involved in producing pro-inflammatory factors in MAPK and JAK (Yu et al., 2009). JGF impeded the expressions of p-JNK1 and p-STAT3 in LPS-stimulated RAW264.7 cells (Figures 7B,D,E). It may be correlated to the reduction of GM-CSF. Moreover, baicalin and wogonin-7-O-glucuronide exhibited similar effects (Supplementary Figure S6), suggesting the potential anti-inflammation mechanism of JGF. Simultaneously, JGF activated slightly the JAK2 and MAPK signals (Figure 5). Future studies should determine the JGF alteration to pSTAT3 downregulation via LPS-stimulated JAK signaling and the potential compound of JGF to activate macrophages. Taken together, the present findings suggested that JGF acts as an immunomodulatory agent and provides a solid anti-inflammation effect. Future studies should further delineate the molecular components of JGF responsible for these dual effects and validate these observations in disease models.

The findings of this study are encouraging, inflammation is a multifaceted and complex process, and thus definitive clinical conclusions cannot be drawn solely based on our data. For the bench-to-bed implications, a converted human dose of 32 mg/kg or lower (from the dose of 400 mg/kg in the mouse model) of this decoction might be considered for use in acute inflammatory conditions in patients. As mentioned, the recommended dose by TCM physicians for JGF oral solution is 30 mg/kg; the difference between the suggested and recommended is likely clinically insignificant. The current concept for antiviral medication is to prevent cell entry and the replication of the virus inside the cell (Kausar et al., 2021). These medications could not 100 percent prevent the virus from spreading nor relieve the hyperinflammation once this phenomenon was presented (Gao et al., 2024; Madewell et al., 2023). Under these circumstances, JGF might be a complementary approach to re-balance this overt state, which was overwhelmed by the uncontrolled production of proinflammatory cytokines (cytokine storm) involving the macrophages (Fajgenbaum and June 2020).

Although JGF is an herbal formulation developed based on traditional Chinese medicine theory and extensive clinical practice, and is generally regarded as having a favorable safety profile, its potential for drug–herb interactions should not be overlooked. Notably, several key constituents of JGF, such as *S*. *baicalensis* (Huang Qin), contain bioactive compounds like baicalin that have been shown to inhibit cytochrome P450 enzymes, including CYP3A4 (Zhou et al., 2021). This suggests a possible risk of metabolic interactions when co-administered with conventional pharmaceuticals. Moreover, the immunomodulatory properties of JGF—particularly its ability to suppress IL-6, STAT3, and GM-CSF signaling pathways—may interfere with corticosteroids, immunosuppressive agents, or cytokine-based therapies. While direct clinical interactions between JGF and standard medications have rarely been reported to date, a recent study demonstrated that co-administration of JGF with nirmatrelvir resulted in elevated systemic drug levels in animal models (Lee et al., 2024). These findings suggest a potential adjuvant role for JGF in enhancing drug exposure. Nevertheless, the mechanisms underlying herb–drug interactions involving JGF and its individual botanical components remain largely unexplored. Further investigation, particularly studies focusing on pharmacokinetics and clinical safety, is warranted to comprehensively assess the interaction risks associated with JGF use.

## Conclusion

This study demonstrates that JGF functions as a bidirectional immune modulator, despite its partial inhibition of macrophage viability at high concentrations. Under basal conditions, JGF activates macrophages by enhancing the production of NO and IL-6, primarily through the upregulation of iNOS, COX-2, JAK2, and MAPK signaling pathways, as observed in RAW264.7 and MH-S cell lines. In contrast, under LPS-induced inflammatory conditions, JGF selectively suppresses JNK1 signaling, thereby attenuating STAT3-mediated expression of iNOS and COX-2. Furthermore, *in vivo* experiments revealed that while JGF alone may mildly induce certain pro-inflammatory mediators, it markedly mitigates LPS-induced acute inflammation in mice. Collectively, this study provides the first evidence of the immunomodulatory effects of JGF on macrophages, supporting its potential as a novel herbal intervention for inflammation control.

## Data Availability

The original contributions presented in the study are included in the article/Supplementary Material, further inquiries can be directed to the corresponding author.

## Abbreviations

ACE2, Angiotensin-converting enzyme-2; *COX-2, Cyclooxygenase-2*; COVID-19, The coronavirus disease 2019; DMEM, Dulbecco’s Modified Eagle Medium; FBS, Fetal Bovine Serum; iNOS, Inducible nitric oxide synthase; JGF, Jing Guan Fang; LPS, Lipopolysaccharide; JAK2, Janus kinase 2; MTT, 3-(4,5-Dimethylthiazol-2-yl)-2,5-diphenyltetrazolium bromide; SARS-CoV-2, Severe acute respiratory syndrome coronavirus 2; STAT3, Signal transducer and activator of transcription 3; TCM, Traditional Chinese medicine.
