# Supplementary material for: Jing Guan Fang, an herbal formula, as an immunomodulator: opposing effects on basal and lipopolysaccharide-induced inflammation of macrophage via JAK/STAT3 and MAPK pathways
Source: Front Pharmacol. 2025 Aug 6;16:1618488. doi: 10.3389/fphar.2025.1618488 (PMC12365646; doi:10.3389/fphar.2025.1618488)
Supplement: Supplementary file 1 [file DataSheet1.pdf]

Jing Guan Fang, an herbal formula, as an immunomodulator: opposing effects on basal and lipopolysaccharide-induced inflammation of macrophage via JAK/STAT3 and MAPK pathways

Zhi-Hu Lin, Hsin Yeh, Sang-Nguyen-Cao Phan, Li-Lan Liao,
Chien-Chang Chen, Wei-Hung Hsu, Chung-Hua Hsu, Tung-Yi Lin*

Supplementary information

Supplementary Figure 1

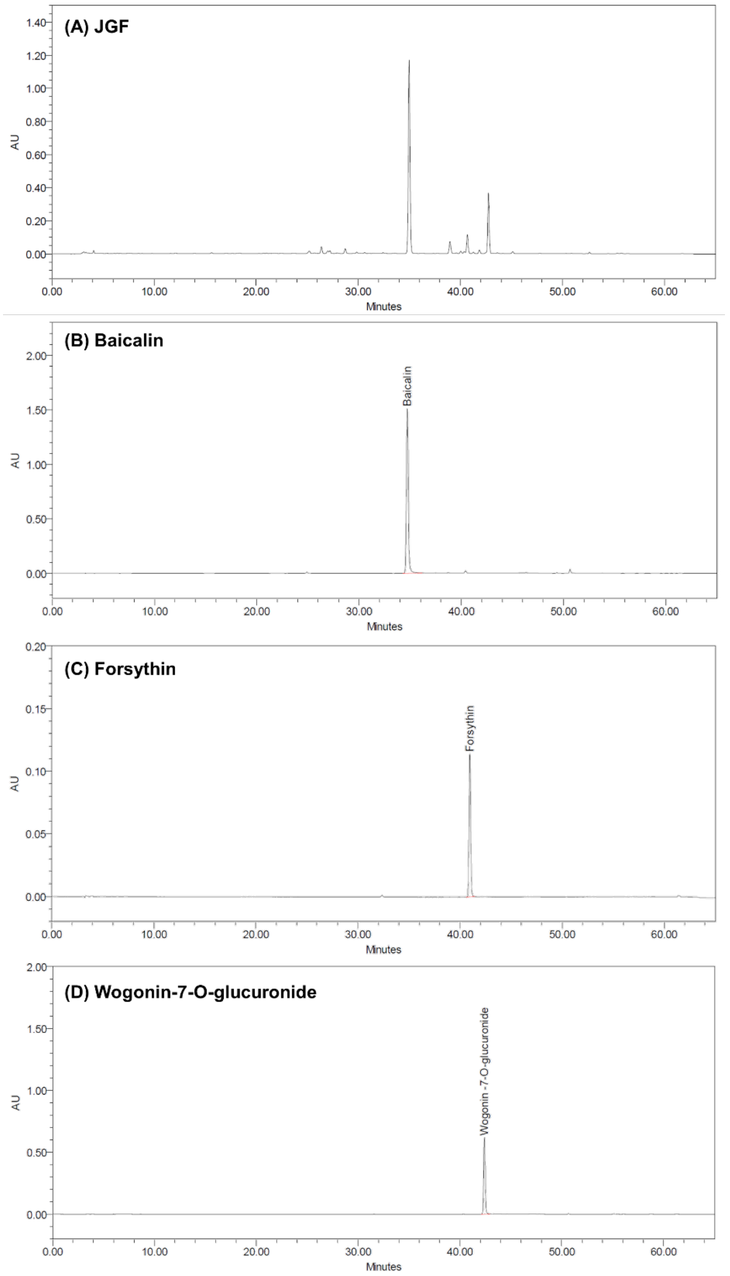

Supplementary Figure 1. The HPLC-UV chromatographic fingerprint profile of JGF and standard compounds at 280 nm.

Supplementary Figure 2

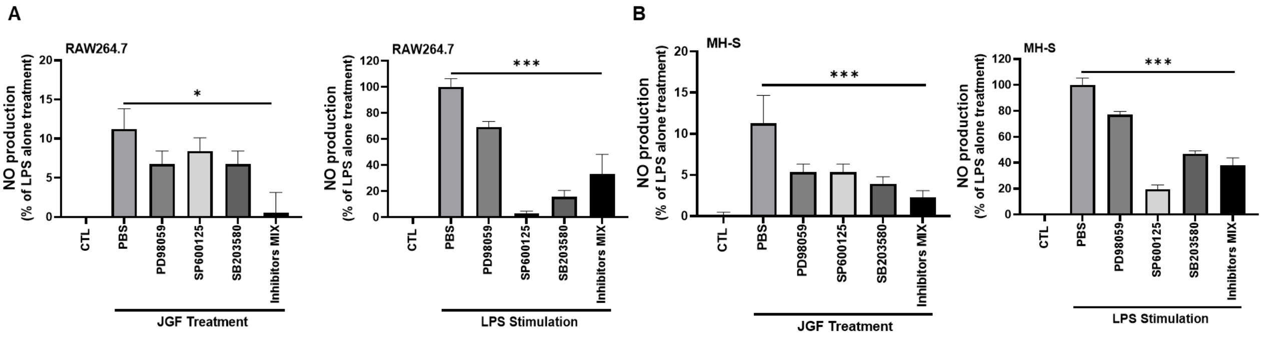

Supplementary Figure 2. MAPK pathway inhibitors suppress JGF- or LPS-induced NO production in RAW264.7 and MH-S macrophages.

(A) RAW264.7 cells and (B) MH-S cells were pretreated with MAPK pathway inhibitors (20 μM, or inhibitor mix) for 30 mins, followed by stimulation with either JGF (600 μg/mL) or LPS (100 ng/mL) for 24 h. NO production in the culture supernatant was measured using the Griess assay and is presented as a percentage of NO levels induced by LPS alone (set as 100%). *$p < 0.05$ compared to JGF or LPS alone group.

Supplementary Figure 3

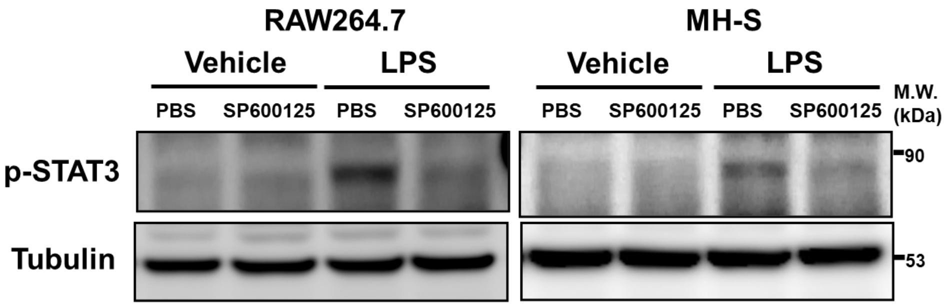

Supplementary Figure 3. SP600125 reduces LPS-induced phosphorylation of STAT3 on macrophages.

RAW264.7 and MH-S cells were stimulated with or without LPS (100 ng/mL) in the presence or absence of JNK inhibitor (SP600125; 10 μM) for 3 h. The phosphorylated levels of STAT3 were determined by Western blot.

Supplementary Figure 4

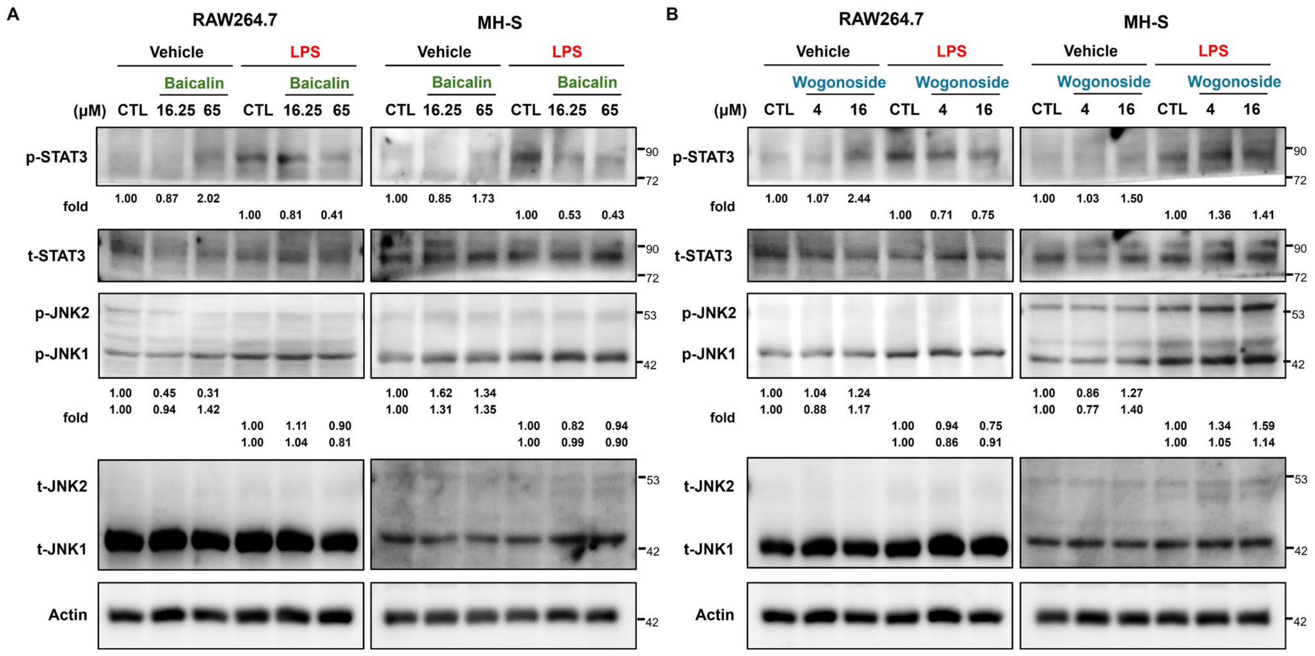

Supplementary Figure 4. Inhibitory effects of baicalin and wogonin-7-O-glucuronide on the phosphorylation of inflammatory molecules in macrophages.

(A) RAW264.7 and MH-S cells were treated with baicalin (16.25 and 65 μM) or **(B)** wogonin-7-O-glucuronide (wogonoside, 4 and 16 μM) and then stimulation with or without LPS (100 ng/mL) for 24 h. Levels of p-STAT3, total-STAT3 (t-STAT3), p-JNK1/2, and total-JNK (t-JNK) were assessed by Western blot. Actin is an internal control. The expression level of each protein was analyzed by ImageJ software.

Supplementary Figure 5

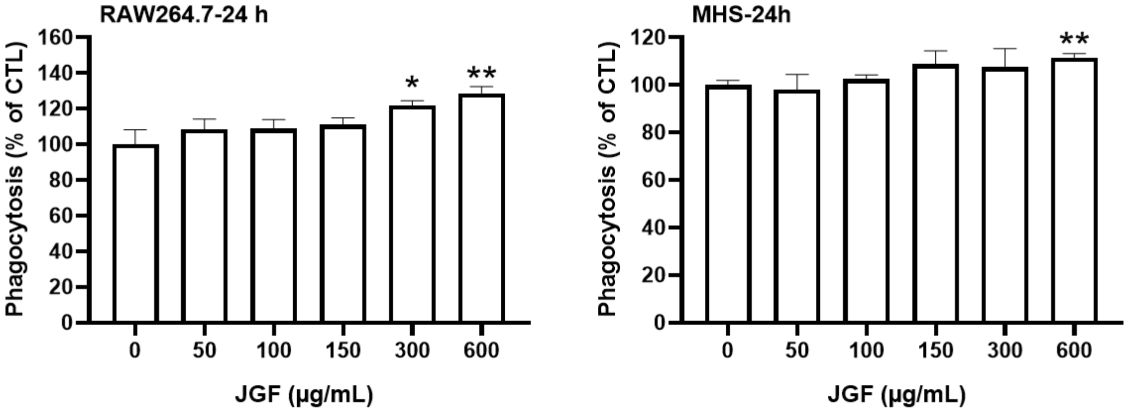

Supplementary Figure 5. JGF slightly increased the phagocytosis of macrophages

RAW264.7 and MH-S cells were treated with various concentrations of JGF (0, 50, 100, 150, 300, and 600 μg/mL) for 24 h. Following, neutral red (Merck; Buchs, St. Gallen, Switzerland) was added to each well at a final concentration of 30 μg/mL and incubated at 37 °C for 1 h. Subsequently, the cells were lysed using a de-staining solution composed of 50% ethanol, 49 % deionized water, and 1% acetic acid. The uptake of neutral red, indicative of cell viability, was quantified by measuring the absorbance at 540 nm.

Supplementary Figure 6

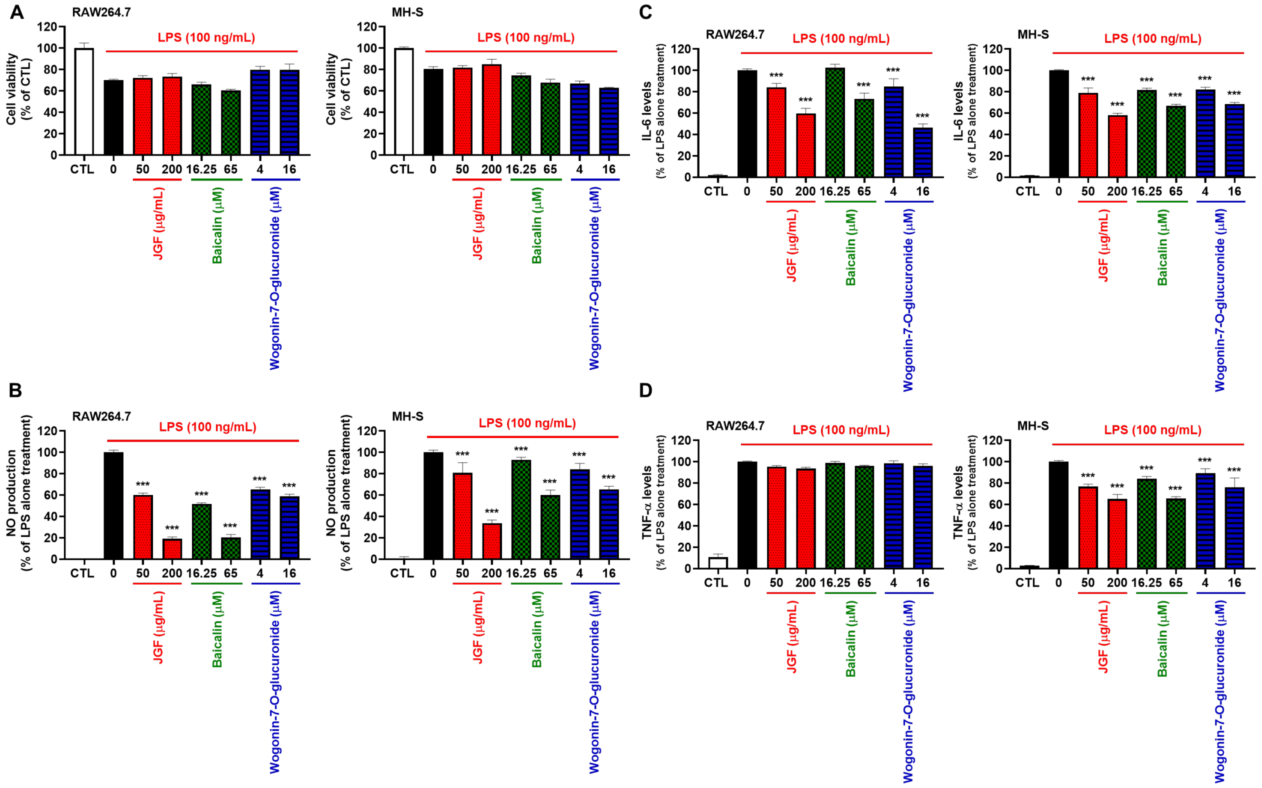

Supplementary Figure 6. The effects of baicalin and wogonin-7-O-glucuronide on inhibition of LPS-induced inflammation on macrophages.

RAW264.7 and MH-S cells were stimulated with LPS (100 ng/mL) in the presence or absence of JGF (50 and 200 μg/mL), baicalin (16.25 and 65 μM), and wogonin-7-O-glucuronide (wogonoside; 4 and 16 μM) for 24 h simultaneously. (**A**) Cell viability was measured by the crystal violet assay. (**B**) NO production was measured by Griess assay. (**C**) IL-6 and (**D**) TNF-α levels were measured by ELISA. The treatment groups with baicalin and wogonoside were normalized relative to the LPS treatment group. Data are representative of three independent experiments and are expressed as mean ± SD, with error bars indicating SD. Significant differences are indicated (***P < 0.001).

Supplementary Table 1. The ratio (JGF/CTL) of various molecules on C3 and C4 cytokine array.

C3 cytokine array

	1	2	3	4	5	6	7	8	9	10	11	12	13	14
1	POS	POS	NEG	NEG	Blank	axl	BLC	CD30L	CD30/TNFRSF8	CD40	CRG-2	CTACK	CXCL16	Eotaxin
2	POS	POS	NEG	NEG	Blank	axl	BLC	CD30L	CD30/TNFRSF8	CD40	CRG-2	CTACK	CXCL16	Eotaxin
**JGF/CTL**						**0.72**	**1.02**	**0.58**	**0.44**	**0.49**	**0.90**	**1.19**	**1.12**	**1.08**
3	Eotaxin-2	FAS ligand	Fractalkine	G-CSF	GM-CSF	IFN-gamma	IGF-BP-3	IGF-BP-5	IGF-BP-6	IL1-alpha	IL1-beta	IL2	IL3	IL3 Rb
4	Eotaxin-2	FAS ligand	Fractalkine	G-CSF	GM-CSF	IFN-gamma	IGF-BP-3	IGF-BP-5	IGF-BP-6	IL1-alpha	IL1-beta	IL2	IL3	IL3 Rb
**JGF/CTL**	**0.59**	**0.64**	**0.59**	**0.66**	**0.98**	**-**	**0.76**	**1.22**	**1.03**	**1.01**	**1.42**	**1.28**	**0.86**	**0.88**
5	IL4	IL5	IL6	IL9	IL10	IL12-p40/p70	IL12-p70	IL13	IL17	KC	Leptin R	Leptin	LIX	L-Selectin
6	IL4	IL5	IL6	IL9	IL10	IL12-p40/p70	IL12-p70	IL13	IL17	KC	Leptin R	Leptin	LIX	L-Selectin
**JGF/CTL**	**0.65**	**0.40**	**0.44**	**0.50**	**0.67**	**0.50**	**0.20**	**0.56**	**0.01**	**0.84**	**0.89**	**-**	**0.80**	**0.99**
7	Lymphotactin	MCP-1	MCP-5	M-CSF	MIG	MIP-1-alpha	MIP-1-gamma	MIP-2	MIP-3-beta	MIP-3-alpha	PF4	P-Selectin	RANTES	SCF
8	Lymphotactin	MCP-1	MCP-5	M-CSF	MIG	MIP-1-alpha	MIP-1-gamma	MIP-2	MIP-3-beta	MIP-3-alpha	PF4	P-Selectin	RANTES	SCF
**JGF/CTL**	**0.63**	**0.38**	**0.16**	**0.62**	**-**	**0.67**	**0.76**	**0.78**	**0.61**	**0.10**	**0.73**	**0.65**	**0.46**	**-**
9	SDF-1-alpha	TARC	TCA-3	TECK	TIMP-1	TNF-alpha	sTNF RI	sTNF RII	TPO	VCAM-1	VEGF	Blank	Blank	POS
10	SDF-1-alpha	TARC	TCA-3	TECK	TIMP-1	TNF-alpha	sTNF RI	sTNF RII	TPO	VCAM-1	VEGF	Blank	Blank	POS
**JGF/CTL**	**0.61**	**0.56**	**0.78**	**0.55**	**0.70**	**0.66**	**0.34**	**0.58**	**0.70**	**0.70**	**1.82**			

Footnote: In the table, the background color represents different JGF/CTL ratio ranges: pink indicates a ratio greater than or equal to 1.25; yellow indicates a ratio between 0.8 and 1.25; and blue indicates a ratio less than or equal to 0.67. A (-) symbol signifies that the cytokine array was unable to detect the target.

C4 cytokine array

	1	2	3	4	5	6	7	8	9	10	11	12
1	POS	POS	NEG	NEG	Blank	bFGF	DPPIV/CD26	Dtk	E-Selectin	Fcg RIIB	Flt-3 Ligan	GITR
2	POS	POS	NEG	NEG	Blank	bFGF	DPPIV/CD26	Dtk	E-Selectin	Fcg RIIB	Flt-3 Ligan	GITR
**JGF/CTL**						**0.75**	**-**	**1.22**	**-**	**0.37**	**0.48**	**0.90**
3	HGF R	ICAM-1	IGFBP-2	IGF-I	IGF-II	IL-15	IL-17B R	IL-7	I-TAC	Lungkine	MDC	MMP-2
4	HGF R	ICAM-1	IGFBP-2	IGF-I	IGF-II	IL-15	IL-17B R	IL-7	I-TAC	Lungkine	MDC	MMP-2
**JGF/CTL**	**-**	**0.61**	**0.77**	**1.13**	**0.58**	**-**	**-**	**-**	**0.68**	**0.68**	**0.10**	**-**
5	MMP-3	Osteopontin	Osteoporotegerin	Pro-MMP-9	Resistin	Shh-N	Thymus CK-1	TIMP-2	TRANCE	TROY	TSLP	VEGF R1
6	MMP-3	Osteopontin	Osteoporotegerin	Pro-MMP-9	Resistin	Shh-N	Thymus CK-1	TIMP-2	TRANCE	TROY	TSLP	VEGF R1
**JGF/CTL**	**5.24**	**0.59**	**-**	**0.20**	**2.15**	**-**	**-**	**0.56**	**-**	**-**	**-**	**0.20**
7	VEGF R2	VEGF R3	VEGF-D	Blank	Blank	Blank	Blank	Blank	Blank	Blank	Blank	POS
8	VEGF R2	VEGF R3	VEGF-D	Blank	Blank	Blank	Blank	Blank	Blank	Blank	Blank	POS
**JGF/CTL**	**0.63**	**0.10**	**-**									

Footnote: In the table, the background color represents different JGF/CTL ratio ranges: pink indicates a ratio greater than or equal to 1.25; yellow indicates a ratio between 0.8 and 1.25; and blue indicates a ratio less than or equal to 0.67. A (-) symbol signifies that the cytokine array was unable to detect the target.

Supplementary Table 2. The ratio [(JGF+LPS)/LPS] of various molecules on C3 and C4 cytokine array.

C3 cytokine array

	1	2	3	4	5	6	7	8	9	10	11	12	13	14
1	POS	POS	NEG	NEG	Blank	axl	BLC	CD30L	CD30/TNFRSF8	CD40	CRG-2	CTACK	CXCL16	Eotaxin
2	POS	POS	NEG	NEG	Blank	axl	BLC	CD30L	CD30/TNFRSF8	CD40	CRG-2	CTACK	CXCL16	Eotaxin
**{(JGF+LPS)/LPS}**						**0.86**	**0.82**	**0.83**	**-**	**0.77**	**0.49**	**1.28**	**1.05**	**0.63**
3	Eotaxin-2	FAS ligand	Fractalkine	G-CSF	GM-CSF	IFN-gamma	IGF-BP-3	IGF-BP-5	IGF-BP-6	IL1-alpha	IL1-beta	IL2	IL3	IL3 Rb
4	Eotaxin-2	FAS ligand	Fractalkine	G-CSF	GM-CSF	IFN-gamma	IGF-BP-3	IGF-BP-5	IGF-BP-6	IL1-alpha	IL1-beta	IL2	IL3	IL3 Rb
**{(JGF+LPS)/LPS}**	**0.46**	**0.70**	**0.81**	**1.51**	**0.11**	**-**	**0.72**	**0.80**	**0.79**	**0.92**	**1.04**	**1.02**	**1.08**	**0.87**
5	IL4	IL5	IL6	IL9	IL10	IL12-p40/p70	IL12-p70	IL13	IL17	KC	Leptin R	Leptin	LIX	L-Selectin
6	IL4	IL5	IL6	IL9	IL10	IL12-p40/p70	IL12-p70	IL13	IL17	KC	Leptin R	Leptin	LIX	L-Selectin
**{(JGF+LPS)/LPS}**	**0.69**	**0.47**	**0.79**	**0.69**	**1.80**	**0.94**	**0.95**	**0.78**	**-**	**0.99**	**0.82**	**-**	**1.02**	**0.83**
7	Lymphotactin	MCP-1	MCP-5	M-CSF	MIG	MIP-1-alpha	MIP-1-gamma	MIP-2	MIP-3-beta	MIP-3-alpha	PF4	P-Selectin	RANTES	SCF
8	Lymphotactin	MCP-1	MCP-5	M-CSF	MIG	MIP-1-alpha	MIP-1-gamma	MIP-2	MIP-3-beta	MIP-3-alpha	PF4	P-Selectin	RANTES	SCF
**{(JGF+LPS)/LPS}**	**0.62**	**1.20**	**0.54**	**0.92**	**-**	**1.21**	**1.29**	**1.10**	**1.09**	**0.53**	**2.26**	**1.58**	**0.92**	**-**
9	SDF-1-alpha	TARC	TCA-3	TECK	TIMP-1	TNF-alpha	sTNF RI	sTNF RII	TPO	VCAM-1	VEGF	Blank	Blank	POS
10	SDF-1-alpha	TARC	TCA-3	TECK	TIMP-1	TNF-alpha	sTNF RI	sTNF RII	TPO	VCAM-1	VEGF	Blank	Blank	POS
**{(JGF+LPS)/LPS}**	**0.63**	**0.64**	**0.95**	**0.82**	**-**	**0.14**	**0.93**	**1.14**	**0.94**	**1.03**	**3.47**			

Footnote: In the table, the background color represents different [(JGF+LPS)/LPS] ratio ranges: pink indicates a ratio greater than or equal to 1.25, yellow indicates a ratio between 0.8 and 1.25, and blue indicates a ratio less than or equal to 0.67. A (-) symbol signifies that the cytokine array was unable to detect the target.

C4 cytokine array

	1	2	3	4	5	6	7	8	9	10	11	12
1	POS	POS	NEG	NEG	Blank	bFGF	DPPIV/CD26	Dtk	E-Selectin	Fcg RIIB	Flt-3 Ligan	GITR
2	POS	POS	NEG	NEG	Blank	bFGF	DPPIV/CD26	Dtk	E-Selectin	Fcg RIIB	Flt-3 Ligan	GITR
**{(JGF+LPS)/LPS}**						**1.61**	**-**	**1.21**	**-**	**1.95**	**0.94**	**1.06**
3	HGF R	ICAM-1	IGFBP-2	IGF-I	IGF-II	IL-15	IL-17B R	IL-7	I-TAC	Lungkine	MDC	MMP-2
4	HGF R	ICAM-1	IGFBP-2	IGF-I	IGF-II	IL-15	IL-17B R	IL-7	I-TAC	Lungkine	MDC	MMP-2
**{(JGF+LPS)/LPS}**	**-**	**1.06**	**1.11**	**0.75**	**1.11**	**-**	**1.03**	**-**	**1.00**	**1.13**	**0.20**	**1.10**
5	MMP-3	Osteopontin	Osteoprotegerin	Pro-MMP-9	Resistin	Shh-N	Thymus CK-1	TIMP-2	TRANCE	TROY	TSLP	VEGF R1
6	MMP-3	Osteopontin	Osteoprotegerin	Pro-MMP-9	Resistin	Shh-N	Thymus CK-1	TIMP-2	TRANCE	TROY	TSLP	VEGF R1
**{(JGF+LPS)/LPS}**	**2.09**	**1.13**	**-**	**0.22**	**-**	**-**	**-**	**0.89**	**1.06**	**-**	**-**	**0.50**
7	VEGF R2	VEGF R3	VEGF-D	Blank	Blank	Blank	Blank	Blank	Blank	Blank	Blank	POS
8	VEGF R2	VEGF R3	VEGF-D	Blank	Blank	Blank	Blank	Blank	Blank	Blank	Blank	POS
**{(JGF+LPS)/LPS}**	**0.71**	**0.83**	**-**									

Footnote: In the table, the background color represents different [(JGF+LPS)/LPS] ratio ranges: pink indicates a ratio greater than or equal to 1.25; yellow indicates a ratio between 0.8 and 1.25; and blue indicates a ratio less than or equal to 0.67. A (-) symbol signifies that the cytokine array was unable to detect the target.

Supplementary Table 3. Major constituents and their immunomodulatory effects of mornach, minister and assistant herbs

Herb	Major component	Immunomodulation properties	References
**Lianqiao *(Fructus Forsythiae)***	Forsythiasides	Regulation of TLR4/MyD88)/NF-κB, and Nrf2/HO-1	(1)
	Forsythoside A	Regulation of autophagy and apoptosis through the AMPK/mTOR/ULK1 pathway	(2)
		Inhibition of p38 JNK/MAPK/ERK and NF-κB signaling	(3)
		Inhibition of inflammation and epithelial barrier damages in lung and colon through PPAR-γ/RXR-α complex	(4)
**Huangqin *(Radix Scutellariae baicalensis)***	Baicalin	Modulation of inflammatory response of macrophages activated by LPS via calcium-CHOP Pathway.	(5)
		Enhancement of macrophage efferocytosis via inhibition of RhoA/ROCK signaling pathway and regulating macrophage polarization.	(6)
		Inhibition of LPS-induced inflammation in RAW264.7 cells through miR-181b/HMGB1/TRL4/NF-κB pathway.	(7)
	Wogonoside	Inhibition of inflammatory cytokine production in LPS-stimulated macrophages via JNK/c-Jun signaling pathway.	(8)
**Chaihu (*Radix Bupleuri*)**	Saikosaponin A	Inhibition of the MAPK and NF-κB pathways in LPS-stimulated RAW264.7 cells	(9), (10)
		Suppression of LPS-induced NF-κB activation and NLRP3 inflammasome expression	(11)

Notes: 5'-monophosphate-activated protein kinase (AMPK), calcium/cAMP-response element-binding protein homologous protein (Calcium-CHOP), cell adhesion molecules (CAMs), high mobility group box 1 (HMGB1), heme oxygenase-1 (HO-1), Jun N-terminal kinase (JNK), lipopolysaccharide (LPS), mechanistic target of rapamycin (mTOR), microRNA-181b (miR-181b), mitogen-activated protein kinase

(MAPK), myeloid differentiation factor 88 (MyD88), nuclear factor kappaB (NF-κB), nuclear factor-erythroid 2–related factor 2 (Nrf2), NOD-, LRR- and pyrin domain-containing protein 3 (NLRP3), peroxisome proliferator–activated receptor gamma (PPAR-γ), Ras homolog family member A (RhoA), retinoid X receptor alpha (RXR-α), RAW264.7 murine macrophage cell line (RAW264.7), Rho-associated coiled-coil containing protein kinase (ROCK), Toll-like receptor 4 (TLR4), unc-51 like autophagy activating kinase 1 (ULK1).

References

1. Yang HX, Liu QP, Zhou YX, et al. Forsythiasides: A review of the pharmacological effects. Front Cardiovasc Med. 2022. 9:971491.
2. Zhang X, Zhang H, Gao Y, et al. Forsythoside A regulates autophagy and apoptosis through the AMPK/mTOR/ULK1 pathway and alleviates inflammatory damage in MAC-T cells. Int Immunopharmacol. 2023. 118:110053.
3. Song L, Lei Y. Forsythoside A inhibited inflammatory response by inhibiting p38 JNK/MAPK/ERK and NF-κB signaling in Staphylococcus aureus pneumonia. J Mol Histol. 2025. 56(3):147.
4. Wang J, Xue X, Zhao X, et al. Forsythiaside A alleviates acute lung injury by inhibiting inflammation and epithelial barrier damages in lung and colon through PPAR-γ/RXR-α complex. J Adv Res. 2024. 60:183-200.
5. An HJ, Lee JY, Park W. Baicalin Modulates Inflammatory Response of Macrophages Activated by LPS via Calcium-CHOP Pathway. Cells. 2022.11(19):3076.
6. Cai X, Shi Y, Dai Y, Wang F, Chen X, Li X. Baicalin clears inflammation by enhancing macrophage efferocytosis via inhibition of RhoA/ROCK signaling pathway and regulating macrophage polarization. Int Immunopharmacol. 2022. 105:108532.
7. Yan G, Chen L, Wang H, Wu S, Li S, Wang X. Baicalin inhibits LPS-induced inflammation in RAW264.7 cells through miR-181b/HMGB1/TRL4/NF-κB pathway. Am J Transl Res. 2021. 13(9):10127-10141.
8. Yu X, Chen D, Wang L, et al. Wogonoside inhibits inflammatory cytokine production in lipopolysaccharide-stimulated macrophage by suppressing the activation of the JNK/c-Jun signaling pathway. Ann Transl Med. 2020. 8(8):532.
9. Zhu J, Luo C, Wang P, He Q, Zhou J, Peng H. Saikosaponin A mediates the inflammatory response by inhibiting the MAPK and NF-κB pathways in LPS-stimulated RAW 264.7 cells. Exp Ther Med. 2013. 5(5):1345-1350.
10. Yuan B, Yang R, Ma Y, Zhou S, Zhang X, Liu Y. A systematic review of the active saikosaponins and extracts isolated from Radix Bupleuri and their applications. Pharm Biol. 2017. 55(1):620-635.
11. Du ZA, Sun MN, Hu ZS. Saikosaponin a Ameliorates LPS-Induced Acute Lung Injury in Mice. Inflammation. 2018. 41(1):193-198.
